# Editorial Chit Chat

**Published:** 1869-07

**Authors:** 


### EDITORIAL CHIT CHAT.

**Another Want.**

A new name for trout. "Speckled beauties" is about "played out," as our urchins say.

**A Novelty.**

A patent has been obtained by a New York gentleman, for a water proof paper. Our grocers will soon be sending molasses and vinegar to their customers, done up in paper bags.

**Large Bequest.**

The Hartford papers say that Mrs. Harriet Hosmer, recently deceased, left $45,000 to the Hartford Hospital, and an additional $25,000 on condition that homœopaths be recognized.

**Large Prize to Inventors.**

The Brazilian Government has offered a reward of $8,000 in gold to the inventor of any practical mode of preserving beef for exportation. More than four hundred experiments have been made, but none have succeeded.

**A Good Law.**

The Legislature of Illinois has passed a law classifying drunkards with idiots and insane people, and providing guardians to take charge of their property and persons.

Truly a sensible law. Hope we may have just such an one in our own state.

**A Want.**

Why don't some enterprising Masonic Publishing House give us a directory of all the Masonic bodies in the United States? Stating their time of meeting, &c. It would be useful in every Lodge, Chapter, Commandery and Consistory in the Union. Hope some one will take the hint, for which we make no charge.

**Oblige Us.**

When our readers have occasion to address any of our advertisers, with reference to their business, they will oblige us by saying that they saw the advertisement in the BISTOURY. This will be but little trouble and will satisfy our patrons that they get the worth of their money.

**Looks Badly.**

An advertising sheet, called the "*Crystal Springs Theraputist*," is wholly copied from Dr. Gleason's "*Physiologist*," of Philadelphia. Why don't the Crystal Spring folks secure the services of a physician, and one who can write his own articles?

**Cheeky.**

To ask us to reply to a half dozen queries so that you can *borrow* the BISTOURY of a neighbor and read our answer. We answer *subscribers*, or those who inclose their fifty cents, and become so, first; if we have space and inclination, we then reply to "dead heads."

**A Nuisance.**

The wool-tallow-hide-bees-wax-coon-skin-buying establishment, just east of the Railroad bridge, on Water street. The stench emanating from this building is intolerable. A store-house of this character should not be permitted on so populons a thoroughfare. Will not our city fathers see that this nuisance is abated?

**Credit.**

"We thank Mr. Stivers of the Orange County *Press* for copying one of our editorials, entitled "Publication of Crime." It would have been still more pleasing to us had he given due credit for it."—*Waverly Advocate*.

And equally pleasing to *us*, Mr. *Advocate*, had you given *us* credit for the article on "Swindlers," which you copied from the BISTOURY, some time ago.

**A Common Disease.**

"A young girl of thirteen, (says a Paris paper,) Miss Dumesuil, is said to be imbued with a fluid of an extraordinary attractive power, which attracts all objects of wood which surround her. Chairs and tables are instantly attracted toward her when she approaches near them."

We have frequently heard cases of the above character in our own city, where the parties "imbued with a fluid of extraordinary attractive power," have been *cured* by a six month's imprisonment in our county jail.

**Academy of Sciences.**

This institution is now in a flourishing condition. Monthly meetings are held, learned essays read by the members, and a good time had generally. We were highly entertained by a most scholarly production, from the pen of Prof. Ford, on "Flower Coloring," at the last monthly meeting.

The Academy building is being rapidly filled with specimens in natural history, and these, together with the fine telescope and other astronomical paraphanalia, renders the place of great interest to the visitor.

**Answers to Queries.**

Parties writing us, and requiring answers "by return mail," must not forget to inclose a stamp to pay return postage. Do not inclose a prepaid envelope. They seldom fit our business paper. We have stationery which suits us pretty well, and we prefer to use it. Parties who desire advice, upon medical subjects, through the BISTOURY, should not forget to enclose fifty cents and become subscribers; they will then have some claim upon us, and will be more apt to receive a reply.

**Quack Indians.**

Of all the numerous quacks who perambulate the country none are more thoroughly imposters than those who claim to be "Indian Doctors." The Indians of this country were never acquainted with the virtue of the most common herbs, and when attacked by a formidable disease died unless nature and cold water, which they did use to some extent, allowed a cure. Their medicine men were the offspring of superstition and acted as a kind of priest to perform heathen incantations and attempt cures by mummery. These who palm themselves off as having learned the art of healing disease from ignorant savages, are imposters and quacks.

**Excellent Lectures.**

Those given by Mr. Beecher at the Opera House, every Sunday evening during the past year. Our large Opera House has been crowded, and hundreds have been unable to obtain seats, upon every occasion of Mr. Beecher's preaching.

The warm weather has caused a discontinuance of the meetings for the present. There will be rejoicings among the people, we are sure, when they are again resumed.

Mr. Beecher has introduced the novelty of leading the singing for his immense congregation, with a brass band, and if you desire to hear the best congregational singing in this country, go to the Sunday meetings at the Opera House, this fall—listen—be pleased and instructed.

**Has He Called?**

That two hundred or so, avordupois chap, who has just returned from Jerusalem with *only* one set of handsome photographs, shaded in India-ink, of the "four seasons" &c. Offers to sell them for the very low figure of $25, but will take the insignificant sum of eight dollars if he can't get more. He tells you "they are the only copies of the work in this country," and that "you never saw anything like it before." If you tell him another fellow called last week with the same "works," and the same story, he strokes his beard and replies, "O, yes, I sends 'im here."

Seen him? When he calls, as he certainly will, tell him he's an old humbug and a worse liar, and that his works are not worth more than one dollar apiece, and that you can buy cart-loads of them at that figure, in New York—and you'll tell him the truth in every instance.

---

**Fun with Bismuth.**

If we write with a pen dipped in a solution of the nitrate of bismuth, after it is dry nothing can be seen, but as soon as we plunge the paper in water, the writing will become distinctly visible. Secret intelligence can be conveyed in this manner by writing between the lines of ink with the solution of bismuth. Lovers with watchful and distrustful parents will take the hint at once.

A mixture of two parts of bismuth, one of lead, and one of tin, will melt at 200° Fahrenheit. Spoons can be cast of this alloy for the purpose of playing tricks upon your friends at a tea-party. The moment the victim of the trick commences to stir his tea, the spoon melts away and disappears in the bottom of the cup, leaving a blunt handle in his hand to confound him before the company.

---

**Nonsense.**

To urge a fellow to eat when he does'nt want to. "*Do* have another piece of steak—have some more strawberries—let me help you to something more, can't I?"—"Why, you don't make out *half* a meal—*do* have another warm biscuit, I made them myself, and I *know* they are nice." Such and similar expressions one will meet with at almost every house he chances to take a friendly meal. Persons *will* urge visitors to eat, even after they have stoutly refused two or three times, until one *must* eat in order to get rid of the coaxing.

We took just such a teasing (and the victuals too) the other evening, and suffered with headache all next day, just to please our friend, but we inwardly cursed him, every time our temples gave a throb and our stomach indulged in a twist.

Don't *urge* your visitors to eat; it is confounded bad manners, and you are rendering them liable to headache and dyspepsia, two afflictions for which we are sure they will not thank you.

---

**A New Dodge.**

We have received numerous propositions from so-called advertising agencies, in which we have been invited to publish certain cards, and to send our charges to their office for collection *after* we had done the work. The parties who are engaged in this business, being entirely irresponsible, expect, doubtless, to make a "nice thing" out of their system of free advertising. But by far the greatest jokers of them all is a concern calling themselves "Abbott & Company's Bureau for General Advertising," who send us the following modest little card:—

"*Publisher of the* BISTOURY.—Please insert the accompanying advertisement of the ——— Sewing Machine, as long as you can afford, to be paid as follows: On receipt of your paper BY US, containing *marked copy* of such advertisement, we will mail to you the due bill of the ——— Sewing Machine Co., which will be good for the No. 1 Machine, Price $60, upon the payment of $30 in cash and surrender of the due bill.

Very Respectfully,

ABBOTT & CO."

Now, isn't that "cool?" Pay them thirty dollars in cash, and publish their card beside, for a worthless machine that we wouldn't have in the house. Try again, Messrs. Abbott & Co. BISTOURY is a little too sharp to bite at such a bait as that.

The "Eugenie Man. Co.," are another comical set of chaps. They want us to publish a series of advertisements for which they will forward us "a very useful article for every lady," the retail price of which is $2.50.

Doubtless, their article is of value to every female, but we desire to assure the "Eugenie Co.," that we have no use for the article whatever, and that $2.50 would not pay for one of their small cards, one insertion.

---

**Fifth Ward School.**

We imagine that very few of our neighbors in the Fifth Ward are aware of the excellence of the public school in that ward. Recently we visited the school and were delighted with the splendid discipline of the scholars, and the thorough course of drill which they have had in their various studies. Mr. PARLEY COUBORN

evidently "knows how to teach school," and if he had a suitable building for his scholars, we are sure he could have, if indeed he has not already, the model school of the city.

Some of the rooms contain entirely too many scholars—eighty-four were packed in a room about 15 x 30 feet. It is criminal to jeopardize the lives of our little ones in this manner. The rooms are illy constructed, having but little means of ventilation, and the crowded condition of the seats, where the children are compelled to sit snugly together, scarcely giving them room for the inflation of their lungs, is a state of affairs that should not be allowed to exist in this enlightened age. It is absolutely an unhealthy and unsafe place to send children. Rather let them go without schooling, than to have them inhale the seeds of disease in this miserable school-room.

We learn that a suitable site has been purchased for a new school house in the Fifth Ward, but that the Board of Education are afraid to ask for a new building until they are fairly out of debt for those built in other parts of the city. Then let us have no schools held in this ward until we *can* have a suitable building in which to hold them. Better by far have ignorant children than unhealthy ones; for no child can attend school daily in some of the present rooms of the school and escape disease. This fact will be evident to any intelligent person who will take the trouble to visit the place during school hours as we have done.

Give Mr. Couburn a new school house and he will repay you in good discipline, and render your children more healthful and happy.

